# Adaptation and Reach of a Pre-Exposure Prophylaxis Social Marketing Campaign for Latino, Latina, and Latinx Populations: Development Study

**DOI:** 10.2196/52842

**Published:** 2024-07-17

**Authors:** Harita S Shah, Pedro Alonso Serrano, Gregory Phillips II

**Affiliations:** 1 Department of Medicine University of Chicago Chicago, IL United States; 2 Department of Medical Social Sciences Northwestern University Chicago, IL United States; 3 Department of Preventive Medicine Northwestern University Chicago, IL United States

**Keywords:** Latino, Latinx, Latina, social marketing, social media, PrEP, pre-exposure prophylaxis, HIV prevention, community, CBPR, community-based participatory research, campaign, transgender, MSM, reach, HIV, prevention, formative research, men who have sex with men, treatment, intervention, biomedical, awareness, Latino/x/a, Latina/x/o

## Abstract

**Background:**

Latino, Latina, and Latinx (Latino/a/x) individuals remain disproportionately impacted by HIV, particularly sexual minority men and transgender women. Pre-exposure prophylaxis (PrEP) is an effective means of biomedical HIV prevention, but awareness and uptake remain low among marginalized Latino/a/x populations. Social marketing campaigns have demonstrated promise in promoting PrEP in other populations but are poorly studied in Latino/a/x sexual minority men and transgender women.

**Objective:**

This study aims to (1) adapt and pilot a PrEP social marketing campaign tailored to Latino/a/x populations with a focus on sexual minority men and transgender women through community-based participatory research (CBPR) and (2) evaluate the reach and ad performance of the adapted PrEP social marketing campaign.

**Methods:**

We used the ADAPT-ITT (assessment, decision, adaptation, production, topical experts-integration, training, and testing) framework for adapting evidence-based interventions for new settings or populations. This paper presents how each phase of the ADAPT-ITT framework was applied via CBPR to create the PrEPárate (“Be PrEPared”) campaign. Key community engagement strategies included shared ownership with community partners, focus groups to guide content, crowdsourcing to name the campaign, design by local Latino/a/x artists, and featuring local influencers as the faces of PrEPárate. We evaluated campaign reach and advertisement performance using social media platform metrics (paid and organic reach, impressions, unique clicks, and click-through rates [CTR]) and website use statistics from Google Analytics.

**Results:**

The PrEPárate campaign ran in Cook County, Illinois, from April to September 2022. The campaign reached over 118,750 people on social media (55,750 on Facebook and Instagram [Meta Platforms Inc] and 63,000 on TikTok [ByteDance Ltd]). The Meta ads performed over the industry benchmark with ads featuring local transgender women (2% CTR) and cisgender sexual minority men (1.4% CTR). Of the different Grindr (Grindr Inc) ad formats piloted, the interstitial Grindr ads were the highest performing (1183/55,479, 2.13% CTR). YouTube (Google) ads were low performing at 0.11% (153/138,337) CTR and were stopped prematurely, given limits on sexual education–related content. In the first year, there were 5006 visitors to the website.

**Conclusions:**

Adaptation of an existing evidence-based intervention served as an effective method for developing a PrEP social marketing campaign for Latino/a/x audiences. CBPR and strong community partnerships were essential to tailor materials and provide avenues to systematically address barriers to PrEP access. Social marketing is a promising strategy to promote PrEP among underserved Latino/a/x populations.

## Introduction

### Disparities in HIV Prevention Impacting Latino, Latina, and Latinx Populations

Despite tremendous strides in HIV prevention and treatment, Latino, Latina, and Latinx (Latino/a/x) populations remain disproportionately impacted by HIV. In 2019, Latino/a/x individuals represented approximately 18% of the US population but accounted for 29% of the 34,801 new HIV diagnoses [1]. The majority of Latino/a/x persons diagnosed with HIV were sexual minority men (76% of those diagnosed). Latina transgender women similarly are impacted by disparities in HIV outcomes, with 35% of Latina transgender women having HIV compared to 17% of White transgender women [2]. Following national trends, Latino/a/x individuals in Chicago, Illinois, face higher HIV incidence and progression to AIDS than their non-Latino/a/x White peers [3]. Chicago is embedded within Cook County, 1 of the 48 “hotspot” jurisdictions with the highest burden of HIV prioritized in the US Ending the HIV Epidemic initiative, and it is 1 of the 7 jurisdictions driving the epidemic among Latino/a/x populations specifically [4,5].

Pre-exposure prophylaxis (PrEP) is an effective biomedical intervention to prevent HIV infection among populations vulnerable to HIV [5]. PrEP is available as an oral pill or long-acting injection and has been shown to be safe and effective in preventing HIV infection, particularly among sexual minority men and transgender women at elevated risk for HIV [6,7]. While the uptake of PrEP has increased overall, specific groups (young sexual minority men, transgender women, and racial or ethnic minority populations) have been less likely to initiate and adhere to PrEP [8-10]. Similar to trends across the United States, Latino/a/x sexual minority men and transgender women in Chicago have been shown to have disproportionately low uptake of PrEP relative to their White counterparts [11].

Latino/a/x sexual minority men and transgender women are distinct and diverse populations, yet they also face overlapping barriers related to intersectional identities that contribute to disparities in PrEP uptake and HIV incidence. Lack of awareness is a critical barrier for PrEP uptake among Latino/a/x individuals, particularly among immigrants and those with limited English proficiency [12-14]. While US-born Latino/a/x sexual minority men and transgender women have greater awareness of PrEP, knowledge gaps remain in terms of PrEP modalities (oral pill vs long-acting injection), how to access PrEP, PrEP effectiveness, and potential side effects [15]. Latino/a/x sexual minority men and transgender women face further barriers to PrEP related to homophobia or biphobia, transphobia, racism, and discrimination at multiple levels (eg, interpersonal, community, and structural) [15-18]. Additional structural barriers vary by location but often include lack of health insurance, language discordance, limited health literacy, and immigration status [12-14].

In studies of PrEP interest and acceptability, once they become aware of PrEP including the long-acting injection, Latino/a/x sexual minority men and transgender women reported willingness to adopt PrEP at the same or higher levels than their non-Latino/a/x peers [12,19]. These studies revealed that improving awareness of PrEP is key to reducing disparities in PrEP uptake among Latino/a/x individuals. It is critical that interventions to increase PrEP awareness for Latino/a/x sexual minority men and transgender women be tailored through community-based participatory research (CBPR) to concurrently address barriers to PrEP uptake [5,9,20,21].

### Promise of Social Marketing Campaigns for PrEP Promotion

Social marketing campaigns present a promising opportunity to promote PrEP among Latino/a/x populations. “Social marketing” involves the application of commercial marketing principles to design and implement programs to effect health behavior change, often through social media and offline advertisements [22]. Social marketing campaigns have been shown to increase PrEP awareness and uptake among Black sexual minority men and transgender women by leveraging peer networks to share information beyond the reach of traditional medical settings [23-25]. One such campaign was the PrEP4Love campaign, a sex-positive campaign that increased PrEP awareness and uptake among Black sexual minority men, transgender women, and cisgender women in Chicago, Illinois [25,26]. However, there is a dearth of literature on PrEP social marketing interventions tailored to Latino/a/x individuals. A few PrEP campaigns have been developed for Black and Latino/a/x sexual and gender minority populations jointly, although these groups have cultural differences that are often better served by separately tailored interventions [15,24]. Research is needed to identify best practices in developing and evaluating Latino/a/x-centered PrEP campaigns.

Social marketing interventions have demonstrated effectiveness in improving other HIV-related outcomes among Latino/a/x sexual minority men and transgender women (eg, condom use and HIV testing) and thus offer considerable potential to promote PrEP [13,23-29]. Latino/x sexual minority men report social media and dating apps as 2 of the main methods in which they have heard about PrEP [12,18]. With 74% of Latino/a/x individuals reporting the internet and social media as their trusted source of information, social marketing presents a relevant means of facilitating PrEP awareness and uptake among Latino/a/x sexual minority men and transgender women, particularly given increased social media use during the COVID-19 pandemic [30]. In this research, we studied the adaptation and reach of a pilot PrEP social marketing campaign for Latino/a/x populations with a focus on sexual minority men and transgender women, based on community input and needs.

## Methods

### Development of Community Partnerships

Following the success of PrEP4Love in 2016, there was significant community interest in a Latino/a/x-centered PrEP campaign in Chicago. This interest led to the formation of the Chicago Queer Latinx (CQL) Collaborative. The CQL Collaborative is a group of 10 Latino/a/x and LGBTQ+ (lesbian, gay, bisexual, transgender, queer) stakeholders representing community-based organizations (CBOs) that provide HIV services to Latino/a/x individuals. In 2018, the CQL Collaborative partnered with the Illinois PrEP Working Group to identify best practices to adapt PrEP4Love for a Latino/a/x audience through mixed methods [31,32]. This foundational work was followed by community-public-academic partnerships between the CQL Collaborative and investigators at the University of Chicago, Northwestern University, and Cook County Health to develop, implement, and evaluate a Latino/a/x-centered PrEP social marketing campaign. Following the principles of CBPR, the CQL Collaborative members were equal partners in this initiative since its inception [21,33].

### Adaptation of PrEP4Love

We used the ADAPT-ITT framework (assessment, decision, adaptation, production, topical experts-integration, training, and testing), which is recommended for adapting evidence-based interventions in HIV research for new settings or populations (Table 1) [34]. Through consensus with the CQL Collaborative, we chose to focus on sexual minority men and transgender women within a range of Latino/a/x sexual and gender identities so as not to stigmatize a certain group. While Latino/a/x sexual minority men and transgender women are distinct and diverse groups, we chose to prioritize both given the urgent need to address HIV incidence in these Ending the HIV Epidemic priority populations and given the certain aforementioned shared barriers to PrEP uptake. Thus, this pilot intervention was tailored to address those common barriers and inform future work that could be more robustly tailored to distinct subgroups.

**Table 1:** Process of adapting a pre-exposure prophylaxis social marketing campaign for Latino/a/x^a^ sexual minority men and transgender women in Cook County, Illinois, via the ADAPT-ITT^b^ framework.

Phase	Timeline	Methodology used for the PrEPárate campaign
Assessment	2018-2019	Focus groups, stakeholder interviews, and surveys with Latino/a/x sexual minority men and transgender women were conducted
Decision	2020	We decided to adapt the evidence-based intervention PrEP4Love for Latino/a/x sexual minority men and transgender women in Cook County, Illinois
Adaptation	2021	The campaign name was selected through a crowdsourcing open contest We contracted 4 local Latino/a/x designers to develop campaign materials We “theatre tested” different design schemes with Latino/a/x sexual minority men and transgender women over Instagram (Meta Platforms Inc) and with CQL^c^ Collaborative members
Production	2022	We arranged photo and video sessions with 7 local Latino/a/x sexual minority men and transgender women social media “influencers” (people with large social media followings among our target audience) We developed a website [35] We contracted a social media agency to create and manage advertisements
Topical experts	2018-2022	The CQL Collaborative was involved in every phase of the project including the review of all campaign materials and study design
Integration	February-April 2022	We integrated feedback from CQL Collaborative members and a survey of Latino/a/x LGBTQ+^d^ youth to refine campaign materials prior to dissemination
Training	January-February 2022	The investigative team completed training in running and evaluating social media advertisements through the Center for AIDS Research Digital Bootcamp The social media agency and web developer were already trained in their respective fields
Testing	April 2022	We tested campaign advertisements in April 2022 and launched the full pilot intervention from May to July 2022 using social media (including dating apps), posters at Latino/a/x-focused CBOs^e^ and clinics, and train and bus advertisements

^a^Latino/a/x: Latino, Latina, and Latinx.

^b^ADAPT-ITT: assessment, decision, adaptation, production, topical experts-integration, training, and testing.

^c^CQL: Chicago Queer Latinx.

^d^LGBTQ+: lesbian, gay, bisexual, transgender, queer.

^e^CBO: community-based organization.

The assessment phase consisted of focus groups, stakeholder interviews, and surveys with Latino/a/x sexual minority men and transgender women, which informed the decision phase [31,32]. Participants were recruited through partner CBOs and Chicago stakeholders with experience working with Latino/a/x populations [31,32]. When presented with different campaign examples, stakeholders overall liked PrEP4Love, particularly the empowerment through love and sex positivity and the use of community members as models. They supported the use of advertisements on social media and dating apps to reach young audiences while also advertising on public transportation and at CBOs. Community members also recommended key adaptations such as the use of both English and Spanish messaging and featuring Latino/a/x individuals of varying skin tones to reflect on the diversity within Latino/a/x identities. Participants preferred colorful images in contrast to black and white to reflect the vibrant colors seen in Latino cultures. They also recommended avoiding oversexualized images to minimize sex stigmatization and instead using images of relatable Latino/a/x individuals in everyday scenarios.

As part of the adaptation phase, we held a crowdsourcing open contest to name the campaign through community input. Crowdsourcing open contests are an effective means of soliciting the wisdom of crowds to develop and vet solutions [36,37]. We announced a contest over Instagram (Meta Platforms Inc) and received 194 submissions in 2 weeks. A panel of community experts judged all entries, and the winning name was PrEPárate (“Be PrEPared”). Regarding production, campaign graphics and videos were designed by local Latino/a/x LGBTQ+ artists. We then contracted a social media agency that specializes in LGBTQ+-focused campaigns to pilot-test and run advertisements across platforms. The budget for advertisement design and campaign management by the social media agency was US $30,000. In terms of integration, community input was integrated throughout the campaign development and implementation. We solicited and integrated feedback on these materials from the CQL Collaborative, the artists, and local Latino/a/x youth. Youth feedback involved brief surveys with recruitment from a youth community advisory board and a larger study’s Instagram page (the Keeping it LITE study).

### Measurement of the Reach of the PrEPárate Campaign

We measured the reach of the PrEPárate campaign social media platform metrics of paid reach and organic reach as well as website use statistics [33]. Reach was defined as the number of unique individuals who viewed the advertisement or post. Social media and Grindr (Grindr Inc) measurements of paid reach, meaning the reach of paid advertisements, included impressions (number of times an advertisement is shown), estimated users reached, and advertisement click-through rates (CTR). CTR is a method of assessing advertisement performance by capturing how many times users interact with advertisements; it is defined by the number of clicks an advertisement receives divided by the number of impressions. Social media platforms also provide organic reach data, meaning how many users are shown a post that did not involve paid promotion or boosting. We used Google Analytics to obtain website use statistics including the number of unique users.

### Ethical Considerations

This study was reviewed and deemed exempt by the University of Chicago Institutional Review Board (IRB21-1123). All participation in the formative focus groups, interviews, and surveys was voluntary and responses were deidentified. The data presented are anonymous and aggregated. Focus group participants received a US $40 stipend and interviewees received US $75 for a 1-hour interview. The individuals pictured in the campaign advertisements signed a media release form to allow their image to be published.

## Results

### PrEPárate Campaign

**Figure 1:**
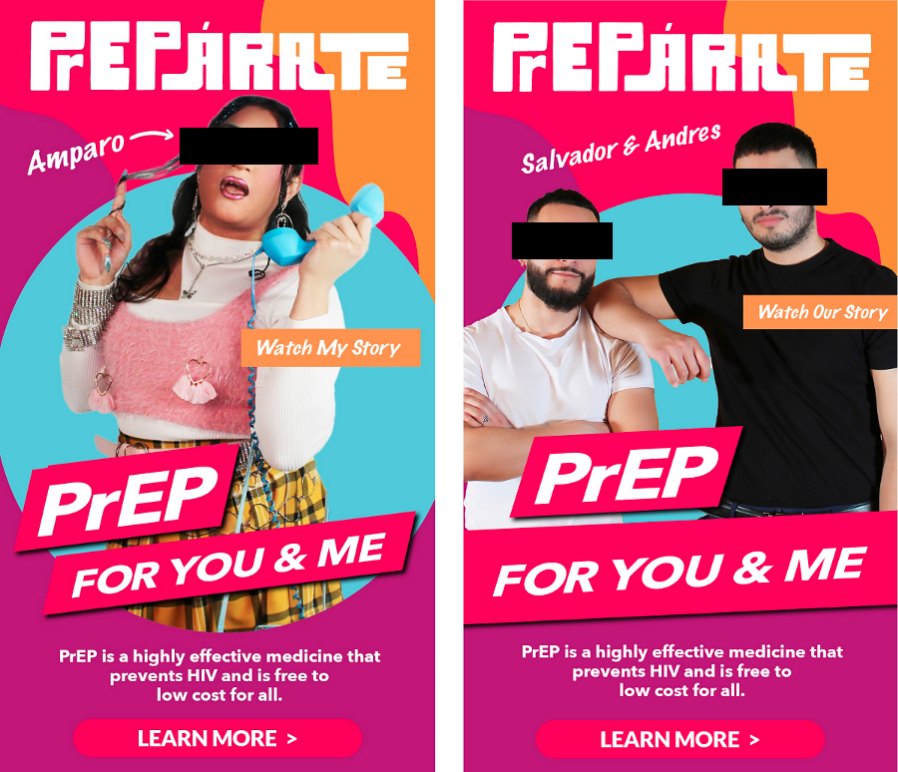
High-performing PrEPárate campaign advertisements for Latino, Latina, and Latinx sexual minority men and transgender women in Cook County, Illinois.

The PrEPárate campaign ran from April to September 2022. Social media and dating app advertisements were piloted in April 2022 to guide the main campaign from May to July 2022. Offline advertisements (eg, bus ads and community events) continued until September 2022. Figure 1 displays the motifs of the PrEPárate campaign. Advertisements featured local Latino/a/x influencers and displayed empowering messaging with bright color themes, as guided by community input [32]. The social media aspect of the campaign included video and graphic content on Facebook and Instagram (Meta Platforms Inc), TikTok, YouTube, and Grindr as guided by Latino/a/x population social media use patterns [38]. We ended up reducing YouTube investment after encountering constraints on targeting and sexual education–related messaging. Social media content included paid advertisements as well as organic posts by community partners and the influencers who were featured in PrEPárate. Advertisements were also distributed on public transit and at community events to reach Latino/a/x individuals who may not have social media access. The call to action of each ad was to visit the PrEPárate website [35]. The website featured information on PrEP and links to access PrEP through the Chicago-based HIV Hub hotline or a web-based PrEP provider tool.

### Reach and Performance of the PrEPárate Social Media Campaign

During the main social media campaign push (from May 10, 2022, to July 31, 2022), the campaign reached approximately 118,750 people over social media. A total of 63,000 individuals were reached on TikTok and 55,750 people were reached on Instagram and Facebook. Additional paid online reach was achieved through Grindr and YouTube advertisements, whose data are presented in the supplementary report on advertisement types and performance (Multimedia Appendix 1). Organic reach was the highest over Instagram reels, with a reach of 19,977 unique users in 1 month compared to Meta’s organic reach of approximately 200 individuals in other months with graphics alone.

The 2 top-performing Meta ads had a CTR of 2%, double the health care industry benchmark of 0.73% [39]. These ads featured well-known Latina transgender women influencers, whose videos were also top performers on Instagram reels and TikTok. The videos celebrated themes of sexual liberation and the use of PrEP to protect the health of them and their partners. The next highest performing ads featured local cisgender Latino/x men and had a CTR of 1.4%. Examples of these top-performing ads are displayed in Figure 1. TikTok ads had a net CTR of 1.03% (2918/283,037), the highest aggregate CTR across platforms. CTR for Grindr ads varied by ad format. We found that full-screen interstitial ads were the highest performing with a CTR of 2.13% (1183/55,479), compared with small banner (472/867,844, 0.05% CTR) and medium banner ads (528/319,787, 0.17% CTR). Preroll YouTube advertisements had a low CTR at 0.11% (153/138,337), and we stopped investment in YouTube ads prematurely given restrictions on sexual education–related content and low performance. Data for these platforms are presented in Multimedia Appendix 1.

In the first year, we received 5006 visitors to the PrEPárate website. A total of 4658 (93%) visitors accessed the website in English, and 4205 (84%) visitors accessed the website on a mobile phone. Approximately half of the users reached the website through social media (2653/5006, 53%) versus the remainder through direct entry of the URL or search (2352/5006, 47%).

## Discussion

### Principal Findings

The PrEPárate campaign was successfully adapted for Latino/a/x sexual minority men and transgender women through strong community engagement and the ADAPT-ITT framework. PrEPárate achieved a reach of over 118,750 people with high-performing social media ads and additional reach over public transit and presence at community events. Community engagement and CBPR were essential to effectively adapting and tailoring the campaign for the target audience. Community engagement strategies that were key to campaign reach included shared ownership with community partners, focus groups to guide content, crowdsourcing to name the campaign, design by local Latino/a/x artists, and featuring local influencers as the faces of PrEPárate.

PrEPárate advertisements with the highest performance featured not only sex-positive language but health-positive language with themes of self-empowerment, consistent with prior literature on PrEP messaging [24-26,40]. Strength-based messaging has been shown to be more effective for HIV prevention efforts than deficit-based messaging, which can perpetuate HIV stigma and PrEP stigma [25,41]. While prior campaigns have emphasized sex-positive content specifically, community input for PrEPárate supported the use of nonsexual content with a goal of celebrating health rather than perpetuating intersectional stigmas surrounding LGBTQ+ identities in various Latino cultures. Future PrEP social marketing interventions should emphasize strength-based messaging and include conscious efforts to avoid stigmatization of priority audiences [15,42].

The PrEPárate campaign was delivered across a variety of online and offline platforms. Among the social media platforms, TikTok had the highest measured reach, correlating with recent trends in which 49% of Latino/a/x adults report TikTok use, greater use than for any other racial or ethnic subgroup [38]. Based on website user language data and the reach data for TikTok (paid) and Instagram reels (organic), the PrEPárate campaign appeared to have the greatest reach to young bilingual or English-speaking Latino/a/x populations. Brief video segments were the most effective means of reaching this young demographic. Our pilot campaign data provide guidance on which platforms and formats to direct advertisement investment for future PrEP campaigns to reach young Latino/a/x audiences. We found TikTok videos, Meta ads featuring certain transgender women and cisgender sexual minority men ambassadors, and Grindr interstitial ads to have the highest performance in terms of CTR. Future directions for PrEPárate may include concerted efforts to reach older Latino/a/x individuals who may be less connected with social media.

While social marketing interventions have the ability to reach national or global audiences, our work supports that tailoring interventions on a local level through CBPR can be advantageous. In wide-scale campaigns, the use of generic models and services can make it feel less credible or relevant. Formative qualitative work with sexual minority men has shown a preference for content grounded in the local community, which directs them to local trusted organizations, as these messages feel more credible [42]. In our experience, featuring local influencers and organizations was key to greater organic sharing of content, which can be more effective in behavior change than direct views alone [23,43]. Partnering with local influencers and organizations also allows for greater community empowerment, a key tenant of effective CBPR. Finally, prior social marketing research has reinforced the importance of physical presence in communities to complement and build trust in campaign messaging, particularly relevant given the impact of mistrust on PrEP uptake [9,44,45].

### Limitations

This formative research has multiple limitations. Social media platforms provided varying degrees of detail for reach data making comparisons difficult. We provided the social media platform data that we had available. We additionally were unable to measure calls to the HIV Hub hotline, which would have been informative in terms of PrEP access and uptake after the campaign, due to hotline staffing and workflow constraints. Finally, while this project provides important formative information on PrEP social marketing campaigns, future rigorous study designs to demonstrate efficacy are needed (eg, cluster-based randomized controlled trial).

### Conclusions

Our formative work on the PrEPárate campaign fills an important gap in PrEP social marketing research for Latino/a/x sexual minority men and transgender women. Community engagement was key to adapting and implementing a PrEP social marketing campaign for Latino/a/x sexual minority men and transgender women, who have unique needs related to intersectional identities. Social marketing presents a promising strategy to promote PrEP among underserved Latino/a/x populations.

## Appendix

Multimedia Appendix 1 Report of advertisement performance.

## Abbreviations

ADAPT-ITT: assessment, decision, adaptation, production, topical experts-integration, training, and testing
CBO: community-based organization
CBPR: community-based participatory research
CQL: Chicago Queer Latinx
CTR: click-through rate
Latino/a/x: Latino, Latina, and Latinx
LGBTQ: lesbian, gay, bisexual, transgender, queer
PrEP: pre-exposure prophylaxis

## References

[1] Centers for Disease Control and Prevention Estimated HIV incidence and prevalence in the United States, 2015–2019. HIV Surveillance Supplemental Report 2021;26(1).

[2] Centers for Disease Control and Prevention (2021). HIV Infection, Risk, Prevention, and Testing Behaviors Among Transgender Women: National HIV Behavioral Surveillance : 7 U.S. Cities, 2019-2020.

[3] Chicago Department of Public Health (2020). HIV+STI Data Report.

[4] McCree DH, Walker T, DiNenno E, Hoots B, Valverde E, Ocfemia MCB, Heitgerd J, Stallworth J, Ferro B, Santana A, German EJ, Harris N (2018). A programmatic approach to address increasing HIV diagnoses among Hispanic/Latino MSM, 2010-2014. Prev Med.

[5] Fauci AS, Redfield RR, Sigounas G, Weahkee MD, Giroir BP (2019). Ending the HIV epidemic: a plan for the United States. JAMA.

[6] Deutsch MB, Glidden DV, Sevelius J, Keatley J, McMahan V, Guanira J, Kallas EG, Chariyalertsak S, Grant RM, iPrEx investigators (2015). HIV pre-exposure prophylaxis in transgender women: a subgroup analysis of the iPrEx trial. Lancet HIV.

[7] Grant RM, Lama JR, Anderson PL, McMahan V, Liu AY, Vargas L, Goicochea P, Casapía M, Guanira-Carranza JV, Ramirez-Cardich ME, Montoya-Herrera O, Fernández T, Veloso VG, Buchbinder SP, Chariyalertsak S, Schechter M, Bekker L, Mayer KH, Kallás EG, Amico KR, Mulligan K, Bushman LR, Hance RJ, Ganoza C, Defechereux P, Postle B, Wang F, McConnell JJ, Zheng J, Lee J, Rooney JF, Jaffe HS, Martinez AI, Burns DN, Glidden DV (2010). Preexposure chemoprophylaxis for HIV prevention in men who have sex with men. N Engl J Med.

[8] Marshall BDL, Mimiaga MJ (2015). Uptake and effectiveness of PrEP for transgender women. Lancet HIV.

[9] Page KR, Martinez O, Nieves-Lugo K, Zea MC, Grieb SD, Yamanis TJ, Spear K, Davis WW (2017). Promoting pre-exposure prophylaxis to prevent HIV infections among sexual and gender minority Hispanics/Latinxs. AIDS Educ Prev.

[10] Strauss BB, Greene GJ, Phillips G, Bhatia R, Madkins K, Parsons JT, Mustanski B (2017). Exploring patterns of awareness and use of HIV pre-exposure prophylaxis among young men who have sex with men. AIDS Behav.

[11] Phillips G, Raman A, Felt D, Han Y, Mustanski B (2019). Factors associated with PrEP support and disclosure among YMSM and transgender individuals assigned male at birth in Chicago. AIDS Behav.

[12] Dolezal C, Frasca T, Giguere R, Ibitoye M, Cranston RD, Febo I, Mayer KH, McGowan I, Carballo-Diéguez A (2015). Awareness of post-exposure prophylaxis (PEP) and pre-exposure prophylaxis (PrEP) is low but interest is high among men engaging in condomless anal sex with men in Boston, Pittsburgh, and San Juan. AIDS Educ Prev.

[13] Shah HS, Grieb SMD, Flores-Miller A, Yenokyan K, Castellanos-Aguirre J, Greenbaum A, Page KR (2021). Sólo se vive una vez: evaluation of a social marketing campaign promoting HIV screening and prevention for immigrant latinxs. AIDS Behav.

[14] Rao S, Mulatu MS, Xia M, Wang G, Song W, Essuon A, Patel D, Eke A, German EJ (2021). HIV preexposure prophylaxis awareness and referral to providers among Hispanic/Latino persons—United States, 2019. MMWR Morb Mortal Wkly Rep.

[15] Barreras JL, Linnemayr SL, MacCarthy S (2019). "We have a stronger survival mode": exploring knowledge gaps and culturally sensitive messaging of PrEP among Latino men who have sex with men and Latina transgender women in Los Angeles, CA. AIDS Care.

[16] Brooks RA, Nieto O, Landrian A, Donohoe TJ (2019). Persistent stigmatizing and negative perceptions of pre-exposure prophylaxis (PrEP) users: implications for PrEP adoption among Latino men who have sex with men. AIDS Care.

[17] Ogunbajo A, Storholm ED, Ober AJ, Bogart LM, Reback CJ, Flynn R, Lyman P, Morris S (2021). Multilevel barriers to HIV PrEP uptake and adherence among Black and Hispanic/Latinx transgender women in Southern California. AIDS Behav.

[18] Murray A, Gaul Z, Sutton MY, Nanin J (2018). "We hide…": perceptions of HIV risk among Black and Latino MSM in New York City. Am J Mens Health.

[19] Schoenberg P, Edwards OW, Merrill L, Martinez CA, Stephenson R, Sullivan PS, Jones J (2023). Willingness to use and preferences for long-acting injectable PrEP among sexual and gender minority populations in the southern United States, 2021-2022: cross-sectional study. J Int AIDS Soc.

[20] Rodriguez-Diaz CE, Martinez O, Bland S, Crowley JS (2021). Ending the HIV epidemic in US Latinx sexual and gender minorities. Lancet.

[21] Rhodes SD, Malow RM, Jolly C (2010). Community-based participatory research: a new and not-so-new approach to HIV/AIDS prevention, care, and treatment. AIDS Educ Prev.

[22] Andreasen AR (1995). Marketing Social Change: Changing Behavior to Promote Health, Social Development, and the Environment.

[23] Marshall B, Salabarría-Peña Y, Johnson W, Moore L (2022). Reaching racial/ethnic and sexual and gender minorities with HIV prevention information via social marketing. Eval Program Plann.

[24] Kudrati SZ, Hayashi K, Taggart T (2021). Social Media and PrEP: a systematic review of social media campaigns to increase PrEP awareness and uptake among young Black and Latinx MSM and women. AIDS Behav.

[25] Phillips G, Raman A, Felt D, McCuskey DJ, Hayford CS, Pickett J, Lindeman PT, Mustanski B (2020). PrEP4Love: the role of messaging and prevention advocacy in PrEP attitudes, perceptions, and uptake among YMSM and transgender women. J Acquir Immune Defic Syndr.

[26] Dehlin JM, Stillwagon R, Pickett J, Keene L, Schneider JA (2019). #PrEP4Love: an evaluation of a sex-positive HIV prevention campaign. JMIR Public Health Surveill.

[27] Solorio R, Norton-Shelpuk P, Forehand M, Montaño D, Stern J, Aguirre J, Martinez M (2016). Tu amigo pepe: evaluation of a multi-media marketing campaign that targets young Latino immigrant MSM with HIV testing messages. AIDS Behav.

[28] Olshefsky AM, Zive MM, Scolari R, Zuñiga M (2007). Promoting HIV risk awareness and testing in Latinos living on the U.S.-Mexico border: the Tú No Me Conoces social marketing campaign. AIDS Educ Prev.

[29] Patel VV, Ginsburg Z, Golub SA, Horvath KJ, Rios N, Mayer KH, Kim RS, Arnsten JH (2018). Empowering with PrEP (E-PrEP), a peer-led social media-based intervention to facilitate HIV preexposure prophylaxis adoption among young Black and Latinx gay and bisexual men: protocol for a cluster randomized controlled trial. JMIR Res Protoc.

[30] Flores A, Lopez M (2018). Among U.S. Latinos, the internet now rivals television as a source for news. Pew Research Center.

[31] Gilliland I (2019). Summary of Focus Group Findings.

[32] Gilliland I (2019). PrEPXAmor Campaign Final Report.

[33] de Vere Hunt I, Linos E (2022). Social media for public health: framework for social media–based public health campaigns. J Med Internet Res.

[34] Wingood GM, DiClemente RJ (2008). The ADAPT-ITT model: a novel method of adapting evidence-based HIV Interventions. J Acquir Immune Defic Syndr.

[35] PrEPárate.

[36] Brabham DC, Ribisl KM, Kirchner TR, Bernhardt JM (2014). Crowdsourcing applications for public health. Am J Prev Med.

[37] Tang W, Ritchwood TD, Wu D, Ong JJ, Wei C, Iwelunmor J, Tucker JD (2019). Crowdsourcing to improve HIV and sexual health outcomes: a scoping review. Curr HIV/AIDS Rep.

[38] Gottfried J (2024). Americans' social media use. Pew Research Center.

[39] Rudan N (2023). Facebook ads benchmarks for your industry. Databox.

[40] Jaiswal J, LoSchiavo C, Meanley S, Hascher K, Cox AB, Dunlap KB, Singer SN, Halkitis PN (2021). Correlates of PrEP uptake among young sexual minority men and transgender women in New York City: the need to reframe "risk" messaging and normalize preventative health. AIDS Behav.

[41] Bekalu MA, Eggermont S (2014). The relative persuasiveness of gain-framed versus loss-Framed HIV testing message: evidence from a field experiment in northwest Ethiopia. J Health Commun.

[42] Goedel WC, Sutten Coats C, Sowemimo-Coker G, Moitra E, Murphy MJ, van den Berg JJ, Chan PA, Nunn AS (2021). Gay and bisexual men's recommendations for effective digital social marketing campaigns to enhance HIV prevention and care continuity. AIDS Behav.

[43] Noar SM (2006). A 10-year retrospective of research in health mass media campaigns: where do we go from here?. J Health Commun.

[44] Shah HS, Dolwick Grieb SM, Flores-Miller A, Greenbaum A, Castellanos-Aguirre J, Page KR (2020). Sólo Se Vive Una Vez: the implementation and reach of an HIV screening campaign for Latinx immigrants. AIDS Educ Prev.

[45] Shah HS, Miller AF, Yang C, Grieb SM, Lipke M, Bigelow BF, Phillips KH, Palomino P, Page KR (2023). A community-engaged social marketing campaign to promote equitable access to COVID-19 services among Latino immigrants. Am J Public Health.

