# Supplementary material for: Adaptation and Reach of a Pre-Exposure Prophylaxis Social Marketing Campaign for Latino, Latina, and Latinx Populations: Development Study
Source: JMIR Form Res. 2024 Jul 17;8:e52842. doi: 10.2196/52842 (PMC11292145; doi:10.2196/52842)
Supplement: Multimedia Appendix 1 [file formative_v8i1e52842_app1.pdf]

COMMANDO
LGBTQ+ ADVERTISING SPECIALISTS

Campaign: Cook County Research - PrEParate April - July 2022 (1)

Data Updated thru: Mar 23, 2023

Month

DISPLAY MEDIA

Campaign	Property	Placement	Status	Clicks	Impressions	CTR
Cook County Research - PrEParate April - July 2022	Grindr Display	Interstitial 320x480	Completed	1,183	55,479	2.13%
Cook County Research - PrEParate April - July 2022	Grindr Display	Banner 320x50	Completed	472	867,844	0.05%
Cook County Research - PrEParate April - July 2022	Grindr Display	Med Rec 300x250	Completed	528	319,787	0.17%
			**Grand total**	**2,183**	**1,243,110**	**0.18%**

SOCIAL MEDIA

Campaign	Property	Placement	Status	Clicks	Impressions	CTR
Cook County Research - PrEParate April - July 2022	Facebook/Instagram	FB/Insta Posts	Completed	896	144,612	0.62%
Cook County Research - PrEParate April - July 2022	TikTok	:15 sec video	Completed	2,918	283,037	1.03%
			**Grand total**	**3,814**	**427,649**	**0.89%**

VIDEO

Placement	Status	Clicks	Impressions	CTR
PreRoll	Completed	153	138,337	0.11%
